# The antibiotic resistance “mobilome”: searching for the link between environment and clinic

**DOI:** 10.3389/fmicb.2013.00138

**Published:** 2013-05-30

**Authors:** Julie A. Perry, Gerard D. Wright

**Affiliations:** ^1^M. G. DeGroote Institute for Infectious Disease Research, McMaster UniversityHamilton, ON, Canada; ^2^Department of Biochemistry and Biomedical Sciences, McMaster UniversityHamilton, ON, Canada

**Keywords:** antibiotic resistance, horizontal gene transfer, environmental resistome, evolution

## Abstract

Antibiotic resistance is an ancient problem, owing to the co-evolution of antibiotic-producing and target organisms in the soil and other environments over millennia. The environmental “resistome” is the collection of all genes that directly or indirectly contribute to antibiotic resistance. Many of these resistance determinants originate in antibiotic-producing organisms (where they serve to mediate self-immunity), while others become resistance determinants only when mobilized and over-expressed in non-native hosts (like plasmid-encoded β-lactamases). The modern environmental resistome is under selective pressure from human activities such as agriculture, which may influence the composition of the local resistome and lead to gene transfer events. Beyond the environment, we are challenged in the clinic by the rise in both frequency and diversity of antibiotic resistant pathogens. We assume that clinical resistance originated in the environment, but few examples of direct gene exchange between the environmental resistome and the clinical resistome have been documented. Strong evidence exists to suggest that clinical aminoglycoside and vancomycin resistance enzymes, the extended-spectrum β-lactamase CTX-M and the quinolone resistance gene *qnr* have direct links to the environmental resistome. In this review, we highlight recent advances in our understanding of horizontal gene transfer of antibiotic resistance genes from the environment to the clinic. Improvements in sequencing technologies coupled with functional metagenomic studies have revealed previously underappreciated diversity in the environmental resistome, and also established novel genetic links to the clinic. Understanding mechanisms of gene exchange becomes vital in controlling the future dissemination of antibiotic resistance.

## INTRODUCTION

Griffith (1928) demonstrated the transformation of non-virulent *Streptococcus pneumoniae* with a heat-stable substance from a virulent strain. The “transforming principle” was later identified as DNA (Avery et al., 1944), and the notion that genetic material can flow both vertically (from parent to offspring), and horizontally (from siblings or peers) in bacteria continues to form the basis of modern-day molecular genetics. Also in 1928, Alexander Fleming discovered the antibiotic penicillin: arguably one of the most important discoveries in modern medicine, but one that is threatened today by the mechanisms discovered by Griffith and Avery. In this review, we highlight recent advances in our understanding of horizontal gene transfer of antibiotic resistance genes in the environment. The development and use of functional metagenomic techniques coupled with advances in sequencing technologies have revealed new diversity in the antibiotic resistome, and also established novel genetic links between the environment and the clinic.

## ON THE ORIGINS OF RESISTANCE: EXPLORING THE ENVIRONMENTAL RESISTOME

The antibiotic “resistome” is the collection of all genes that directly or indirectly contribute to antibiotic resistance, both in the environment and the clinic. Antibiotic-producing bacteria of the genus *Streptomyces* are found abundantly in soils around the globe, and encode resistance genes against their own antibiotics [the so-called “producer hypothesis” (Cundliffe, 1989; Wright, 2007)]. Importantly, these organisms are often multi-drug resistant (D’Costa et al., 2006), which may reflect their ability to produce more than one antibiotic, or be a by-product of their evolution in proximity to antibiotic-producing neighbors in the soil. It is clear that the environmental “resistome” is a substantial source of resistance genes, and it has been theorized that antibiotic-producing organisms in the environment are the source of resistance genes found in clinical pathogens. Davies and colleagues demonstrated almost 40 years ago that the biochemical activity of aminoglycoside resistance enzymes encoded by producing organisms was identical to those found in pathogens (Benveniste and Davies, 1973). However, direct evidence of gene transfer between the environmental resistome and the clinic are rare as resistance genes may undergo many rounds of evolution between the soil and the clinic (Aminov and Mackie, 2007). The most cited examples of recent exchange between the environmental and clinical resistomes include the class-A extended-spectrum β-lactamase CTX-M, found on plasmids carried by major global pathogens and traced to environmental *Kluyvera* spp. (Humeniuk et al., 2002), and the quinolone resistance gene *qnr* (found on a broad-host range conjugative plasmid from a ciprofloxacin-resistant strain of *Klebsiella pneumoniae* and traced to several environmental waterborne species (Poirel et al., 2005a,b; Baquero et al., 2008). Still, the environmental resistome is so vast (and underexplored) that further links to the clinic are sure to surface.

### SAMPLING THE ENVIRONMENT FOR RESISTANCE GENES

Two main approaches are used to study the environmental resistome: culture-based and metagenomic searches. Culture-based approaches involve growing all microorganisms resistant to a given antibiotic in the lab, and subsequently analyzing their genetics and associated biochemistry for resistance determinants. While the major disadvantage to this technique is that it is estimated that only a small fraction (~1%) of environmental organisms are readily grown in the lab (Amann et al., 1995), culture-based approaches allow detailed studies of the genomic context of resistance (including identification of host species, identification of multiple resistance determinants in a single genome, identification of antibiotic production capabilities of the host, etc.) that is not possible using culture-independent methods. Metagenomic studies are performed by culture-independent extraction of total microbial DNA from the environment, and subsequent analysis using either polymerase chain reaction (PCR)-based methods with specific primers or deep sequencing technologies. While a metagenomic approach has the obvious advantage of overcoming culture-based bias, it can be difficult to design oligonucleotide primers with both enough specificity and flexibility to capture sequences with minor deviation from the reference sequence. Metagenomic datasets can be difficult to assemble, and using bioinformatics alone to search for homologs of known resistance determinants may miss novel resistance determinants that are not sufficiently homologous to known genes. Functional metagenomic approaches (in which a metagenomic library is expressed in a heterologous host and screened for resistance) offer significant advantages over culture-based and metagenomic/PCR-based screens and are discussed in detail in the final section of this review.

### NOVEL RESISTANCE DETERMINANTS AS A RESULT OF GENE MOBILIZATION

Perhaps one of the main reasons why resistance genes are missed in traditional metagenomic screens is that genes may not present as resistance genes in their native context. Often, resistance genes are expressed in low copy number in their native host because they form part of a tightly coordinated network (Martinez, 2008). However, if these genes are mobilized into a new genetic context where regulation is absent (or expression is strongly favored), they many function as novel resistance determinants. An excellent example of this type of resistance gene “repurposing” is the efflux pumps belonging to the resistance/nodulation/cell division (RND) family (reviewed in Dantas and Sommer, 2012). This family of small molecule pumps is found ubiquitously in all kingdoms of living organisms, where they function as transporters of toxic compounds. However, when mobilized and expressed at high levels in pathogens like *Campylobacter jejuni, Escherichia coli*, *Salmonella enterica* serotype Typhimurium or *Pseudomonas aeruginosa*, they are capable of conferring high level antibiotic resistance via drug efflux (reviewed in Piddock, 2006a,b; Dantas and Sommer, 2012). Gene mobilization can therefore contribute to the formation (as well as the dissemination) of antibiotic resistance.

## THE ENVIRONMENTAL “MOBILOME”

Bacterial evolution occurs on an accelerated time scale compared to plant and animal species, due to the ease with which genes move between organisms. Gene transfer allows a bacterium to build on existing adaptations in order to invade a new niche or to be more successful in its current niche; the abundance of naturally transformable bacteria, transducing phage, and conjugative elements present in the environment are testament to the importance of horizontal gene transfer to bacterial evolution (Ochman et al., 2000).

### WHAT DRIVES GENE TRANSFER IN THE ENVIRONMENT?

The modern environmental resistome is under selective pressure from human activities such as the use of antibiotics in agriculture, which may influence the composition of the local resistome and lead to gene transfer events. However, rough calculations suggest that 1 g of soil can contain ~580 different species of actinobacteria, which have the genetic capacity to produce 11,600 bioactive small molecules (Wright, 2010). Since these organisms also encode resistance elements to counteract the effects of the molecules they produce, the networks of selection and resistance that exist in soil are vast, and have evolved over millennia. In a carefully controlled study of 30,000-year old Beringian permafrost, D’Costa et al. (2011) showed that genes conferring resistance to β-lactams, tetracyclines, and glycopeptides existed in the environment well before the use of these antibiotics in the clinic. Structural and functional assays on the glycopeptide resistance element VanA demonstrated that the ancient soil resistance determinant was similar to the modern clinical resistance element. While this study showed that the origin of resistance to several natural product antibiotics is in the environment, a quantitative temporal study of resistance genes in agricultural soil from the Netherlands revealed that levels of all resistance genes investigated rose over time, from the pre-antibiotic era (1940s) to the present (Knapp et al., 2010). Moreover, plasmids isolated from pathogenic bacteria pre-dating the antibiotic era do not contain resistance genes (Hughes and Datta, 1983). While resistance to natural product antibiotics is undoubtedly ancient, anthropomorphic factors are clearly contributing to the mobilization, fixation, and dissemination of resistance genes in the present day. Although there is no direct evidence that antibiotics reach concentrations in the soil that approach the highly selective concentrations used therapeutically, antibiotic resistance genes may be necessary if that niche is affected by agricultural animals or humans frequently treated by antibiotics (Wiedenbeck and Cohan, 2011).

### REQUIREMENTS FOR SUCCESSFUL GENE EXCHANGE

Regardless of mechanism or environment, the success of gene transfer depends first and foremost on the proximity of donor and recipient – they must be in physical proximity, and are generally assumed to share the same niche. Genetic exchange communities are variable in taxonomic composition but usually share vector types (plasmid or transposon) and do not contain strong restriction/modification systems such that genetic exchange is more frequent (Wiedenbeck and Cohan, 2011). DNA transferred via transformation is typically short (often the length of one to several genes), which limits the chances a restriction enzyme target sequence will occur, and increases the chances of recombination in deeply divergent bacterial species (Wiedenbeck and Cohan, 2011). Plasmids transferred via conjugation are often able to carry several resistance genes in tandem (reviewed in Barlow, 2009). Once inside the recipient cell, new DNA must replicate autonomously or integrate into the chromosome of the recipient (by homology or via insertion sequences). Recombination is heavily favored if the sequence is flanked by insertion sequences as integration is therefore independent of sequence homology (Vo et al., 2010).

Integrons and transposons are now widely recognized as playing important roles in the dissemination of antibiotic resistance. Integrons are composed of three key elements: the *intI* integrase, a specific recombination site *attI* and a promoter (expertly reviewed in Stalder et al., 2012). Integrons are found chromosomally, on plasmids and transposons, and are found widely distributed in both the clinic and the environment, especially in Gram-negative bacteria. Interestingly, a class 1 integron found in an isolate of *Pseudomonas* from 15,000- to 40,000-year-old Siberian permafrost contained all the elements characteristic of modern-day clinical class 1 integrons, including being located on a transposon and containing an antibiotic resistance gene (*aadA2*, encoding resistance to streptomycin and spectinomycin; Petrova et al., 2011).

The exchange of DNA can occur passively, but may also be actively controlled. Many naturally competent species of *Streptococcus* undergo autolysis to liberate cellular DNA using the same signaling molecule that triggers DNA uptake in the surviving population (Guiral et al., 2006; Perry et al., 2009). Some genera can actively secrete DNA, including *Acinetobacter*, *Alcaligenes*, *Azotobacter*, *Pseudomonas*, *Micrococcus*, *Bacillus*, and *Flavobacterium* (reviewed in Thomas and Nielsen, 2005). On the flip side, bacteria also express defense mechanisms to prevent invasion by foreign DNA elements. Viral infections are prevented at the level of adsorption, injection, or by abortive infection, while restriction–modification systems and the use of sugar-non-specific nucleases target invading nucleic acids. An area of intense research is immunity mediated by clustered regularly interspaced short palindromic repeats (CRISPR)/Cas motifs, present in most archeal and many bacterial genomes (reviewed in Horvath and Barrangou, 2010).

### GENE TRANSFER IN THE SOIL

While gene exchange occurs in all environments, it has been best studied in the soil. The physical properties of soil (temperature, pH, concentration of nutrients and oxygen, etc.), combined with biological factors (diversity, nature, and total microbial biomass) dictate the frequency and nature of gene transfer in this environment (Aminov, 2011). Soil bacteria typically undergo higher rates of gene transfer in areas of higher nutritional content like the rhizosphere, the phyllosphere, decaying plant and animal tissues, and manure-applied soil (van Elsas and Bailey, 2002), and are capable of transformation, transduction, and conjugation.

Transformation is the uptake and incorporation of extracellular DNA from the environment (Chen et al., 2005). Not all bacteria can become “competent” for genetic transformation [although recent reports put the number of naturally transformable strains at more than 60 (Johnsborg et al., 2007)], but both environmental organisms and clinical pathogens use this method of gene acquisition. Environmental bacteria that undergo natural transformation include species of *Pseudomonas* (most studied of which is *P. stutzeri*), species of *Acinetobacter* (notably *A. baylyi)*, and the plant pathogens *Ralstonia solanacearum* and *Xylella fastidiosa* (reviewed in Seitz and Blokesch, 2012). The mineral/particulate composition of soil has been implicated in protecting naked DNA against degradation on several occasions, making transformation a viable method of gene transfer in the environment (Aminov, 2011). Transformation may also play a role in the dissemination of integron-based resistance both in the environment and in the clinic: in a study using the naturally competent environmental organism *Acinetobacter baylyi*, Domingues et al. (2012) found that DNA from integron-carrying strains of *Acinetobacter, Citrobacter, Enterobacter, Escherichia, Pseudomonas*, and *Salmonella* could confer antibiotic resistance as well as transfer integrons and transposons in a 24 h period. Furthermore, the transformation of *Acinetobacter baylyi* occurred with equal efficiency using the supernatants from heat-killed bacterial cultures; DNA purity was not important for transformation. This study is important in that it demonstrates that environmental bacteria have the capacity to acquire and replicate genes from bacteria found in clinical settings; although it does not show an historical association between antibiotic producers and the origins of clinical resistance, it demonstrates the capacity for genetic exchange between these two communities.

Transduction involves the transfer of genes via bacteriophages, and is likely an important mechanism of HGT in the environment. Bacteriophage found in soil display local tropisms, and become highly adapted to the bacteria present in their immediate environment (Vos et al., 2009). Lysogenic phage are present in approximately 30% of cultivable soil bacteria, and estimates of prevalence range from 4 to 68% in culture-independent assessments of soil (Ghosh et al., 2008). Generalized transducing phages can be isolated from *Streptomyces* sp. (Burke et al., 2001), and Ghosh et al. (2008) demonstrated that viral preparations from soil carry hybrid 16S ribosomal ribonucleic acid (rRNA) genes indicative of horizontal transfer and recombination within the community. A study of the fecal virome of swine fed the common agricultural antibiotics carbadox and ASP250 revealed that in-feed antibiotics induced prophages from gut bacteria, and induced population shifts in both the bacterial and viral populations. However, metagenomic sequencing revealed that most viromes harbored few antibiotic resistance genes (0.01% of total reads; Allen et al., 2011). In a study of bacteriophage DNA isolated from environmental water samples (including both urban sewage and river water), Colomer-Lluch et al. (2011) found a relative abundance of *bla*(TEM) and *bla*(CTX-M). Interestingly, transduction has been linked to the transfer of pathogenicity islands and virulence traits more often than resistance genes in the clinic: sub-inhibitory concentrations of ciprofloxacin have been reported to promote the mobilization and transfer of the SaPIbov1 pathogenicity island in *Staphylococcus aureus* via SOS-response-mediated transduction (Ubeda et al., 2005), and fluoroquinolones trigger the expression and dissemination of prophage genes including Shiga toxin in *Escherichia coli* H57:0157, also via SOS (Zhang et al., 2000). Given the enormous diversity of environmental phage and the relatively few viromes that have been sequenced thus far, further metagenomic exploration will provide many more examples of phage-mediated transfer of resistance in both the environment and the clinic.

The most frequent mechanism of horizontal gene transfer for antibiotic resistance genes is on plasmids and integrative conjugative elements (ICEs or conjugative transposons) via conjugation. Conjugative transfer of DNA requires physical contact between donor and recipient cells, and the formation of a pore through which DNA can pass (Thomas and Nielsen, 2005). Plasmids are classified according to their replication and partitioning systems into specific incompatibility (Inc) groups, where two plasmids belonging to the same Inc group cannot co-exist in the same bacterial cell (Shintani et al., 2010b). Antibiotic resistance genes have been found on plasmids belonging to Inc groups P, Q, N, and W, all of which are characterized by a wide host range, including both environmental and pathogenic bacteria (Popowska and Krawczyk-Balska, 2013). Plasmids of subgroup IncP-1 are highly efficient in their ability to spread via conjugation, and are also able to replicate in virtually all Gram-negative bacteria (Shintani et al., 2010a; Popowska and Krawczyk-Balska, 2013). Plasmids belonging to this Inc group often also carry genes conferring resistance against heavy metals (including Ni, Cd, Co, Cu, Hg, Pb, Zn), which can co-select for antibiotic resistance genes. Due to all these factors combined, it has been suggested that spread of multi-drug resistance in soil, water and wastewater treatment plants is mainly due to IncP-1 plasmids (Popowska and Krawczyk-Balska, 2013).

Integrative conjugative elements include all self-transmissible integrative and conjugative elements, regardless of their mechanism of integration or conjugation (Wozniak and Waldor, 2010). Unlike plasmids, however, ICEs must integrate into the chromosome to be maintained. They require little sequence specificity for integration, however, and are therefore considered capable of both intracellular and intercellular transfer (Wozniak and Waldor, 2010). Since ICEs are known carriers of antibiotic resistance genes [the first known mobile element with ICE-like properties was Tn*916* carrying tetracycline resistance (Franke and Clewell, 1981)] and have broad-host range, they are potentially important links between resistance in the environment and the clinic. For example, ICE elements have recently been characterized in the genus *Frankia*, a member of the actinobacteria (Ghinet et al., 2011). Members of the actinobacteria are prolific antibiotic producers, and usually also resistant to multiple antibiotics (D’Costa et al., 2006). Gene exchange across genera on broad-host range plasmids has been demonstrated to occur in nutrient rich environments like the rhizosphere: an IncP-1 plasmid was shown to undergo high frequency conjugal transfer in bacteria belonging to the alpha, beta, and gamma Proteobacteria, as well as to *Arthrobacter* sp., a member of the actinobacteria (Molbak et al., 2007). Several low %G+C conjugative plasmids conferring resistance to sulfonamides were recently discovered in manure-spread soil (Heuer et al., 2009). This study showed not only conjugative transfer to *Escherichia coli* from the soil-based host (postulated to be *Acinetobacter* sp.), but highlights gene transfer in an environment in which the soil mobilome mixes frequently with human and animal microbiota. Furthermore, *Acinetobacter baumannii* is an environmental organism and an emerging clinical pathogen (Howard et al., 2012). Plasmids found in environmental species that can also be found in the hospital setting offer a further potential link between the environment and the clinic.

### DIVERSITY OF ANTIBIOTIC RESISTANCE ELEMENTS IN WATER ENVIRONMENTS

Antibiotic resistant marine bacteria have been found as far as 522 km offshore and at depths as extreme as 8,200 m (reviewed in Aminov, 2011). Not surprisingly, antibiotic resistance and the presence of plasmids in marine bacteria tends to correlate with the degree of pollution in the environment (Aminov, 2011). Marine environments have been studied both *in situ* using metagenomic sampling methods and under laboratory conditions simulating the natural environment, and undergo all three methods of gene exchange like their counterparts in the soil (for an excellent review, see Aminov, 2011). The importance of water environments for gene exchange is that they are mixing grounds for environmental and clinical organisms. In a study examining the diversity of integron-based resistance genes in freshwater floc, Drudge et al. (2012) used a combination of PCR-based amplification (targeting the conserved regions of type I integrons) and microarray-based detection of resistance genes from the amplicons. This protocol allowed the authors to increase the sensitivity of the assay as well as to provide genomic context to resistance. The effluent from wastewater treatment plants is well-studied for similar reasons, as environmental and clinical organisms mix in this environment in the presence of high levels of pollutants like pesticides, detergents, heavy metals, and antibiotics. Szczepanowski et al. (2009) used a PCR-based approach to identify 140 clinically relevant plasmid-based antibiotic resistance genes in the metagenome of a wastewater treatment plant. In a similar study of wastewater, Parsley et al. (2010) used sequence- and function-based metagenomic approaches to identify resistance determinants from bacterial chromosomes, on plasmids and in viral metagenomes found in activated sludge from a treatment plant. Although these authors did not identify as many resistance genes as Szczepanowski et al. (2009), the diversity of sources of resistance elements emphasizes the broad range of mobile genetic elements present in these environments, and the corresponding broad reach of target organisms.

### A WORD ABOUT FITNESS COSTS

Acquiring resistance genes can incur fitness costs, such that a resistance gene may not become fixed in a population unless a positive selective pressure is present (Martinez, 2012). In many soil and water environments, selective pressures are abundant due to regrettable anthropomorphic activities. However, several studies have shown that the introduction of a resistance gene does not necessarily impose a metabolic burden (reviewed in Martinez, 2012). For example, AmpC β-lactamases are usually accompanied by repressors of their expression on *Salmonella* plasmids (Verdet et al., 2000) which minimize their fitness cost. Antibiotic resistance genes are often found in conjunction with other resistance genes, such that selection for one will maintain the others in the population. Heavy metal resistance genes, and genes coding for the production of siderophores, toxin/antitoxin systems, or bacteriocins are often found on the same mobile elements as resistance genes, which become fixed in the population due to the co-selection of their beneficial counterparts, even in the absence of antibiotic selection.

## LINKING THE SOIL TO THE CLINIC

The mobility of resistance genes is well-documented in both the environment and in the clinic, but tangible links between the two remain elusive. The above-mentioned examples of aminoglycoside resistance (Benveniste and Davies, 1973), CTX-M in *Kluyvera* spp. (Humeniuk et al., 2002) and *qnr* in waterborne *Vibrio*, *Shewanella*, and *Aeromonas* (Poirel et al., 2005a,b; Baquero et al., 2008) are conclusive examples of recent exchange between the environmental and clinical resistomes. Comparing the amino acid sequence of the aminoglycoside phosphotransferase APH(3′) between transposons found in Gram-negative and Gram-positive pathogens and from environmental bacteria (*Bacillus circulans* and *Streptomyces fradiae*) also indicates that they have diverged from a common ancestor (Trieu-Cuot and Courvalin, 1986). These examples provide powerful evidence that resistance is able to move from the environment into pathogens, but given the prevalence of resistance genes in environmental reservoirs, certainly more examples of transfer exist?

The acquisition of antibiotic resistance by clinic pathogens from the environment must follow the same rules as all gene transfer events: the most likely place for gene exchange is a niche shared by pathogens and environmental organisms (Wiedenbeck and Cohan, 2011). Sommer et al. (2009) used functional genomics to clone and express DNA from the human microbiome and select for resistance to various antibiotics. The healthy human microbiome is presumably in constant contact with environmental organisms, but is also exposed to pathogenic microorganisms. This approach identified several new resistance factors, but importantly also identified a number of resistance genes harbored in the aerobic gut flora of healthy individuals that are also found in major pathogens (Sommer et al., 2009). Wiedenbeck and Cohan (2011) propose screening for resistance genes in the metagenomes of other organisms from which we frequently acquire pathogens, like agricultural animals, mice, ticks, and mosquitoes, as commensal microorganisms could provide an important link to pathogens.

The most recent evidence for transfer of resistance genes between the environment and the clinic is provided by Forsberg et al*.* (2012). Using an innovating approach, the authors cultured multi-drug resistant Proteobacteria from the soil to enrich for resistance genes, and a metagenomic library was constructed from the enriched cells (Forsberg et al., 2012). This library was transformed into *Escherichia coli*, and selected on media containing 1 or 12 antibiotics representing sulfonamides, aminoglycosides, phenicols, β-lactams, and tetracyclines at inhibitory concentrations. Resistance was detected against all 12 antibiotics, and 110 resistance genes were identified via homology. Of the genes identified, 18 had 100% identity to entries in GenBank, and a further 32 had ≥90% homology. Importantly, 54% of resistance genes identified by this method were previously unknown, and many of which would not have been predicted to encode resistance genes by sequence alone. Moreover, seven sequences were identified with 100% identity to resistance genes in clinical pathogens, including resistance against β-lactams, tetracyclines, aminoglycosides, sulfonamides, and chloramphenicol. Although the authors could not show definitively that these genes originated in soil organisms due to the nature of their metagenomic approach, these results emphasize the importance of the soil resistome regardless of the direction of gene flow (from soil to clinic, or vice versa).

## CONCLUSION

Regardless of methodology, numerous studies have confirmed the fact that antibiotic resistance genes have an environmental reservoir. However, the number and diversity of resistance genes that are found in clinical pathogens is relatively small compared to the diversity in the environment (Martinez, 2012). Why is there such a diversity of resistance genes in the environment? How are they maintained (and what role do they play) outside the clinic? What is the bottleneck preventing transfer of environmental genes to clinical pathogens (and how can we maintain it)? Due to horizontal gene transfer, the microbial world should be considered in a pan-genomic sense, where the selective pressures applied on the environmental microbiome can result in the recruitment and dissemination of resistance genes in clinical pathogens. Mapping both the environmental resistome and the associated mobilome are important steps in slowing the rise of resistant pathogens in the clinic.

## Conflict of Interest Statement

The authors declare that the research was conducted in the absence of any commercial or financial relationships that could be construed as a potential conflict of interest.
